# CD4^+^CD25^+^ Regulatory T Cells in Intracranial Thrombi Are Inversely Correlated with Hemorrhagic Transformation after Thrombectomy: A Clinical-Immunohistochemical Analysis of Acute Ischemic Stroke

**DOI:** 10.1155/2021/3143248

**Published:** 2021-05-17

**Authors:** Li Gong, Xiaoran Zheng, Weiyan Zhang, Zhongwen Shu, Haichao Wang, Qiong Dong, Letao Sun, Xiang Zhang, Yanxin Zhao, Xueyuan Liu

**Affiliations:** ^1^Department of Neurology, Shanghai Tenth People's Hospital, Tongji University, 301# Middle Yanchang Road, Shanghai 200072, China; ^2^Department of Nursing, Huashan Hospital North, Fudan University, Shanghai 200072, China; ^3^Department of Neurosurgery, Shanghai Tenth People's Hospital, Tongji University, 301# Middle Yanchang Road, Shanghai 200072, China; ^4^Gordon F. Derner School of Psychology, Adelphi University, South Avenue, New York 11530, USA

## Abstract

Mechanical thrombectomy is not only effective for managing patients with acute ischemic stroke (AIS), but it also enables a valuable histological analysis of thrombi. Previous studies indicated that regulatory T cells (Treg) adoptive transfer might alleviate the hemorrhagic transformation. However, whether Treg in intracranial thrombi correlates with hemorrhagic transformation after mechanical thrombectomy remains unclear. This study mainly analyzed the colocation of Treg markers in serial thrombus sections stained serially for CD4 and CD25 in groups of hemorrhagic or nonhemorrhagic transformation. Second, to investigate whether these immunohistochemical parameters could provide any additional information beyond hemorrhagic transformation, we compared the overlap between Treg markers among other groups, such as functional outcomes, stroke subtypes, and gender. Our results showed that the number of CD4^+^CD25^+^ Treg cells was lower in the hemorrhagic transformation thrombi than in the nonhemorrhagic group (*p* < 0.001) but there were no significant differences otherwise. The present finding of CD4^+^CD25^+^ Treg cell reductions in thrombi associated with hemorrhagic transformation provides the histological evidence supporting that thromboinflammation might involve in the pathological process of an acute stroke after mechanical thrombectomy.

## 1. Introduction

Mechanical thrombectomy is a proven effective therapy for patients with acute ischemic stroke (AIS) due to large-vessel occlusions [1–5], but hemorrhagic transformation as a severe complication is not rare in survivors after mechanical thrombectomy [6]. Evidence from previous studies suggests that the inflammatory process induced by misdirected activation of the immune system is related to an increased risk of hemorrhagic transformation [7–9], mainly through the interaction between injured endothelium and misdirected immune cells. Thus, the hypothesis of the retrieval manipulation itself associated with hemorrhagic transformation due to the direct endothelial injury or secondary inflammation of thrombus elements is reasonable [10, 11].

Among a variety of immunological events after a stroke, lymphocytes have been identified as the key leukocyte subpopulation driving the neuroinflammatory response and contributing to clinical outcomes [12–14]. Regulatory T cells (Treg) were identified to be a subclass (5-10%) of CD4^+^ T lymphocytes constitutively expressing CD25 marker. CD4^+^CD25^−^ T cells appear to induce the development of autoimmune disease, while CD4^+^CD25^+^ cells tend to inhibit the development of disease [15]. Thus, CD4^+^CD25^+^ Treg cells have been shown to be critical in the regulation of immune homeostasis and inhibiting pathological inflammation such as in case of endothelium injury, consequently providing neuroprotection again stroke attack [10, 11]. Currently, little is known about the direct interaction of hemorrhagic transformation and CD4^+^CD25^+^ Treg cells in human intracranial thrombi, especially among survivors treated with mechanical thrombectomy therapy. Thus, identification of this potential association, through the evaluation of immunohistochemical markers in retrieved clots, may offer valuable opportunities for investigating the mechanism of immune dyshomeostasis for hemorrhagic transformation after mechanical thrombectomy.

## 2. Materials and Methods

### 2.1. Patients and Study Design

This cohort study was conducted at the Department of Neurology of Shanghai Tenth People's Hospital of Tongji University (Shanghai, China). Consecutive patients with AIS were enrolled from June 2017 to November 2019. This study was approved by the Ethics Committee of Shanghai Tenth People's Hospital and conducted in accordance with the Declaration of Helsinki. Written informed consent was obtained from all of participants or their next of kin.

As reported in our own previous study [6], the baseline characteristics (i.e., demographic data, vascular risk factors, and National Institutes of Health Stroke Scale (NIHSS) score on admission), use of intravenous thrombolysis (IVT), revascularization status, stroke subtype, and functional outcomes were recorded. Hemorrhagic transformation was diagnosed using follow-up computed tomography scans by two radiologists (WL. Cai and RL. Zhang) blinded to clinical data, according to the recommendations of the European Cooperative Acute Stroke Study (ECASS) II [16]. Successful recanalization was defined as a thrombolysis in cerebral infarction (TICI) score of 2b–3 in all arteries, while a favorable functional outcome was defined as a modified Rankin Score (mRS) of 0-2 at 3 months. Stroke subtypes were determined in accordance with the Trial of ORG 10172 in Acute Stroke Treatment (TOAST) classification using the following methods: computed tomography or magnetic resonance imaging, digital subtraction angiography, duplex ultrasound, 24-hour electrocardiography, and transthoracic or transesophageal echocardiography. The institutional ethics committee approved this study. Patients were excluded if they were treated with IVT only, did not have thrombus material suitable for histological analysis, had contraindications for IVT, were lacking key outcome data, or had follow-up loss at 3 months. As a result, a total of 91 thrombi were collected and subjected to histological analysis.

### 2.2. Thrombectomy Procedure

Mechanical thrombectomy was performed in accordance with standard operating procedures as published previously [6]. Only the Solitaire AB retrievable stent (Covidien, Irvine, CA, USA) was deployed at the occlusion site and then removed under recommended negative-pressure aspiration [3].

### 2.3. Immunohistochemical Analysis of Thrombus

A total of 91 thrombi from different vascular regions were obtained. The specimens were formalin-fixed and paraffin-embedded, and consecutive 4 *μ*m thick slices were cut. For immunohistochemical staining, the colocalization in consecutive slices was analyzed by serial staining for the following Treg markers: rabbit monoclonal antibody to CD4 (ab133616; dilution, 1/50; Abcam) and rabbit monoclonal antibody to interleukin-2 (IL-2) receptor alpha (ab128955; dilution, 1/50; Abcam).

### 2.4. Staining Quantification

Immunohistochemical sections were scanned and digitized with a slice scanner using a Leica DM500 microscope and digital camera (Leica, Germany). The histological examination was performed without knowledge of the clinical findings by two board-certified pathologists (Feng and Zhang), and all slices on both standard coloration and immunohistochemical staining were analyzed. CD4^+^- and CD25^+^-positive stains were yellow, brown, or tan. Only the overlap of both CD4^+^- and CD25^+^-positive cells in serial sections was identified for CD4^+^CD25^+^ Treg cells and semiquantitatively analyzed using Image J software version 1.48 (National Institutes of Health, Bethesda, MD, USA). Each section was analyzed at the same magnification (200x). Five fields were observed of each slice, and the number of CD4^+^CD25^+^ Treg cells was recorded. Finally, we calculated the mean number of positive cells within each slice.

### 2.5. Statistical Analysis

Baseline characteristics were summarized as means with standard deviation (SD), medians (with range), or frequency counts and proportions. We compared the baseline characteristics, treatment data, and functional outcomes between patients with hemorrhagic transformation and those with nonhemorrhagic transformation using Fisher's exact test (categorical variables) and the Wilcoxon signed-rank test (continuous variables). All statistical analyses were performed using SPSS software version 23.0 (IBM, Armonk, NY, USA). *p* values < 0.05 were considered statistically significant.

## 3. Results

### 3.1. Baseline Clinical and Treatment Characteristics

In this study, a total of 91 thrombi were retrieved from patients and subjected to histological analysis. The clinical characteristics of the 91 patients are shown in Table 1. The main occlusion site was the middle cerebral artery, while vertebrobasilar occlusions accounted for 26.4%. A total of 50 patients (54.9%) underwent bridging thrombectomy, 41 (45.1%) underwent only direct mechanical thrombectomy, and 84 (92.4%) had successful recanalization defined as TICI > 2b–3. According to the TOAST classification, etiology was confirmed as an atherothrombotic cause in 31 patients, cardioembolic origin (atrial fibrillation) in 56, and other in 4 (cancer in 2, dissection in 1, and unknown in 1). At 24 hours, 32 patients developed hemorrhagic transformation after thrombectomy. The 90-day outcome was a favorable prognosis (mRS of 0–2) in 52 patients (57.2%) and death in 15 (16.5%).

### 3.2. Univariate Analysis of Clinical and Immunohistochemical Features between Hemorrhagic Transformation and Nonhemorrhagic Transformation

Next, we compared the baseline data between the hemorrhagic transformation and nonhemorrhagic transformation groups in Table 2. There were no significant intergroup differences in sex, age, NIHSS scores, or vascular risk factors including hypertension, diabetes, and coronary heart disease. The vascular recanalization rate (TICI 2b–3) was substantially but nonsignificantly lower in the hemorrhagic transformation group than in the nonhemorrhagic transformation group (84.4% versus 96.6%, respectively; *p* = 0.092). In the hemorrhagic transformation group, 62.5% of patients were treated by bridging therapy (thrombectomy combined with intravenous recombinant tissue plasminogen activator (rtPA) use), a nonsignificantly higher rate than that of nonhemorrhagic transformation group (54.2%) (*p* = 0.656). Comparison of the 90-day functional outcome and mortality rate of the two groups revealed that the prognosis of the hemorrhagic transformation group was less favorable (53.1.2% versus 59.3%) and the mortality rate was higher (25% versus 11.9%) than that of the nonhemorrhagic transformation group, but the differences were not significant. Immunohistochemical staining results showed that the number of CD4^+^ cells in the hemorrhagic transformation group was significantly lower than that in the nonhemorrhagic transformation group (2 versus 13; *p* < 0.001), and the counts of CD25^+^ cells showed a similar significant intergroup difference (3 versus 11; *p* < 0.001).

### 3.3. Comparison of Treg Markers (CD4, CD25) in Serial Sections between Hemorrhagic and Nonhemorrhagic Transformation Groups

**Figure 1:**
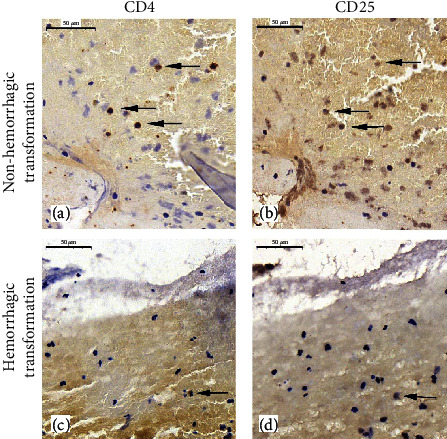
Immunohistochemical staining images of CD4^+^/CD25^+^ Treg cells in serial sections. (a) and (b) are serial images of CD4^+^ and CD25^+^ T cells among the nonhemorrhagic transformation group, respectively. (c) and (d) are serial sections of CD4^+^ and CD25^+^ T cells among the hemorrhagic transformation group, respectively. The dark arrows represent the positive-stained cells. Scale bar: 50 *μ*m.

**Figure 2:**
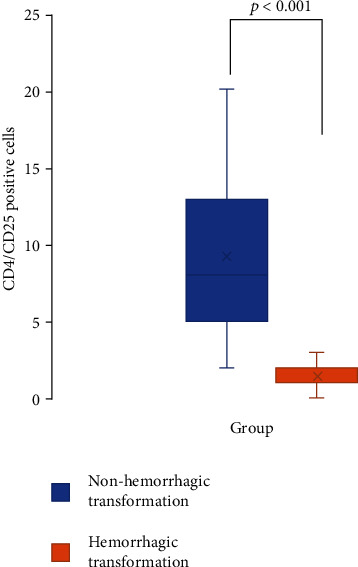
Boxplot showing the relationship of CD4^+^/CD25^+^ Treg cells in intracranial thrombi in the hemorrhagic transformation and nonhemorrhagic transformation groups.

As shown in Figure 1, the analysis of colocalization in serial sections stained serially for CD4 and CD25 showed that there was a minor spatial overlap (Figure 2) of these antibodies in the hemorrhagic transformation group (*p* < 0.001). To investigate whether these serial thrombus sections could provide any additional information beyond hemorrhagic transformation, we further analyzed the colocation of CD4^+^CD25^+^ cells among other groups (Table 3). However, we did not find association of these markers with outcomes, Oxfordshire Community Stroke Project (OCSP) classification, TOAST classification, or gender.

## 4. Discussion

The present observation is based on a serial study of histologically analyzed stroke thrombi retrieved by mechanical thrombectomy [6]. To the best of our knowledge, our results might be the first to show reduction of CD4^+^CD25^+^ Treg cells in human thrombi associated with an increased risk of hemorrhagic transformation after mechanical recanalization.

Emerging evidence suggests inflammation and immune responses in the pathophysiology of stroke [13]. Treg cells play a central role in maintaining immunologic homeostasis, and dysfunction of these cells can induce autoimmune disease. Previous experimental stroke research by Liesz et al. suggests that Treg cells play a neurological recovery function in the chronic phase of stroke [12], as did the results of Mao et al. and Zhou et al. in that these cells can ameliorate intracerebral hemorrhage-induced inflammatory injury and reduce the risk of the hemorrhagic transformation [11, 17]. However, only few studies analyzed Treg cells in stroke survivors, and these studies were limited to the analysis of blood samples from patients in different stages. As reported by Hug et al., circulating Treg function is preserved in the subacute phase after stroke [18], while an opposing finding shows a significant reduction of circulating Treg in stroke patients [19]. In contrast, this finding is not supported by another clinical analysis of Wigren et al. that low levels of circulating Treg cells were not associated with an increased risk of stroke development [20]. These discrepant results can be explained by differing stroke stages, severity, and patient characteristics but also attributed to the reasonable assumption that Treg levels in thrombi might be too confined regionally to reflect that in the circulation. Thus, studies to better analyze Treg cells in human thrombi or plaque after stroke are urgently needed.

In accordance with our finding, Dietel et al. have found that decreased amounts of regulatory T cells from 40 human carotid endarterectomy specimens were associated with vulnerable plaques [21]. In contrast to this result, clinical observations suggested an increased number of Tregs in high-risk atherosclerotic plaques versus stable lesions [22, 23], while the considerable limitation of these studies was the inclusion of stroke-free patients, which might contribute to this diversity. The most commonly used experimental ischemia model is the transient mechanical vascular occlusion (TMVO) model. A prominent characteristic of this model is the occurrence of delayed brain injury due to secondary thromboinflammation [24]. But it was not observed in gradual reperfusion [25]. Thus, thromboinflammation might substantially contribute to pathophysiology in prompt recanalization, particularly in the case of large artery occlusion after mechanical recanalization. Several potential mechanisms resulting in thromboinflammation include the endothelial activation induced by the transient reperfusion, endothelial direct damage by the intravascular manipulation, and the endothelial ischemic injury due to occlusion time [26–28]. Therefore, we assumed that the Treg levels within thrombi, but not in the circulation, might closely reflect the thromboinflammation and thereby the severity of endothelial damage.

A current immunohistochemical analysis of thrombi from acute stroke patients showed a decreased number of CD4^+^ T cells in “white thrombi” versus “erythrocytic thrombi” [23]. As reported in our published study [6], thrombus components with high percentage of fibrin and white blood cells, namely, “white thrombi,” reflect a close association with an atherothrombotic etiology and consequently a high risk of hemorrhagic transformation. Unfortunately, we did not analyze the association between Treg count and white blood cells in retrieved clots in the present study, but it will be needed in future researches. Considering that clot composition might be affected by intravenous rtPA application, we also analyzed the relationship between CD4^+^CD25^+^ cells and rtPA application. We did not observe a significant intergroup difference in Treg counts, suggesting the specific effect of Treg on hemorrhagic transformation after ruling out the rtPA bias.

Our study has several limitations. First, it had a single-center observational design with inherent bias. Second, the retrieved thrombus materials might not always completely reflect those of the entire thrombus; although we tried to obtain sections in the optimal longitudinal plane as representative specimens, this unavoidable bias should be considered. Finally, additional markers for the characterization of Tregs, such as CD127, inducible T cell costimulator, and latency-associated peptide [29, 30], were not tested in the present study. However, these markers have shown controversial results and are under debate; CD4^+^CD25^+^ T cells are still considered the best characterized and representative of Treg cells. The strengths of our study include the clinical characteristics according to various stroke subtypes, rtPA application and functional outcomes, and analysis of immunohistochemical parameters for valuable thrombi from patients after mechanical thrombectomy.

## 5. Conclusion

This clinical study, to the best of our knowledge, is the first to show that levels of CD4^+^CD25^+^ Treg cells in intracranial thrombi inversely correlate with hemorrhagic transformation after mechanical thrombectomy. The group of survivors exhibiting hemorrhagic transformation will benefit from our study if future studies investigate the mechanisms underlying the role of Treg cells from retrieved thrombi.

## Figures and Tables

**Table 1:** Clinical characteristics.

	Histologically analyzed (*N* = 91)
Characteristic	
Age on admission (y), median (range)	70.4 (53-91)
Women, n (%)	44 (48.3%)
Interventional parameters	
TICI score; *n* (%)	
0-2a	7 (7.6%)
2b-3	84 (92.4%)
Bridging therapy, *n* (%)	50 (54.9%)
Occlusion site	
Anterior circulation	67 (73.6%)
Posterior circulation	24 (26.4%)
TOAST, *n* (%)	
Atherosclerosis	31 (34%)
Cardioembolic	56 (61.5%)
Other cause	4 (4.5%)
Vascular risk factors, *n* (%)	
Hypertension	63 (69.2%)
Diabetes mellitus	18 (19.8%)
Coronary heart disease	19 (20.9%)
Hemorrhagic transformation, *n* (%)	32 (35.2%)
Clinical outcome, *n* (%)	
mRS (0-2)	52 (57.2%)
mRS (3–6)	39 (42.8%)
Mortality	15 (16.5%)

TICI: thrombolysis in cerebral infarction; TOAST: Trial of Org 10172 in Acute Stroke Treatment; mRS: modified Rankin Scale.

**Table 2:** Univariate comparison of patients with hemorrhagic versus nonhemorrhagic transformation.

	Hemorrhagic transformation (*n* = 32)	Nonhemorrhagic transformation (*n* = 59)	*p*
Characteristic			
Age on admission (y), median (range)	70 (53-91)	71 (60-87)	0.357
Women, *n* (%)	14 (43.8%)	30 (50.8%)	0.514
NIHSS on admission, median (range)	14 (6-23)	15 (3-22)	0.558
Interventional parameters			
TICI (2b-3 score), *n* (%)	27 (84.4%)	57 (96.6%)	0.092
Bridging therapy, *n* (%)	20 (62.5%)	32 (54.2%)	0.656
TOAST, *n* (%)			0.538
Atherosclerotic	12 (37.5%)	18 (30.5%)	
Cardioembolic	18 (56.3%)	38 (64.4%)	
Other	1 (6.2%)	3 (5.1%)	
Vascular risk factors, *n* (%)			
Hypertension	18 (56.3%)	45 (76.3%)	0.059
Diabetes mellitus	4 (12.5%)	14 (23.7%)	0.272
Coronary heart disease	6 (18.8%)	12 (20.3%)	0.557
Clinical outcome, *n* (%)			0.514
mRS (0-2)	17 (53.1%)	35 (59.3%)	
mRS (3–6)	15 (46.9%)	24 (40.7%)	
Mortality	8 (25%)	7 (11.9%)	0.141
Thrombus components, median (SD)			
CD4^+^ cells^∗^	2 (1.7)	13 (5.6)	<0.001
CD25^+^ cells^∗^	3 (1.5)	11 (7.2)	<0.001

NIHSS: National Institutes of Health Stroke Scale; TICI: thrombolysis in cerebral infarction; TOAST: Trial of Org 10172 in Acute Stroke Treatment; mRS: modified Rankin Scale. ^∗^Primary target variables.

**Table 3:** Relationships of immunohistochemical staining of CD4^+^/CD25^+^ Treg cells with different groups.

	Hemorrhagic transformation^∗∗^		Gender		TOAST		OCSP		Interventional methods		mRS	
	With	Without	Male	Female	LAA	CE	Anterior circulation	Posterior circulation	Bridging therapy	Direct Thrombectomy	0-2	3-6
CD4^+^	2	13	3.5	11.6	8	8.4	8.7	6.0	7.9	8.6	9.8	5.9
CD25^+^	3	11	5.8	11.8	5	7.8	7.8	7.0	5.0	6.5	9.5	4.8
CD4^+^CD25^+^	1.4	8.9	3.2	7.5	4.5	7.8	5	4	3	5.2	5.7	2

TOAST: Trial of Org 10172 in Acute Stroke Treatment; LAA: large atherosclerotic; CE: Cardioembolic; OCSP: Oxfordshire Community Stroke Project; mRS: modified Rankin Scale. ^∗∗^*p* < 0.001.

## Data Availability

The data used to support the findings of this study are available from the corresponding author.
